# Nurse prescribing and dispensing in South Africa: Gaps in the current legislative framework

**DOI:** 10.4102/hsag.v29i0.2582

**Published:** 2024-06-14

**Authors:** Talitha Crowley, Andrew L. Gray, Nelouise Geyer

**Affiliations:** 1 School of Nursing, Faculty of Community and Health Sciences, University of the Western Cape, Cape Town, South Africa; 2 Division of Pharmacology, Discipline of Pharmaceutical Sciences, School of Health Sciences, University of KwaZulu-Natal, Durban, South Africa; 3 Department of Nursing and Education, Faculty of Health, University of the Witwatersrand, Johannesburg, South Africa; 4 Department of Health Studies, Faculty of Human Sciences, University of South Africa, Pretoria, South Africa

**Keywords:** dispensing, legal framework, nurses, prescribing, South Africa

## Abstract

**Background:**

Nurse prescribing and dispensing are central to ensuring universal health access in South Africa.

**Objective:**

To describe the historical development of the legal enablements of nurse prescribing and dispensing in South Africa and highlight gaps in the current legislative framework.

**Method:**

This is a discussion article.

**Results:**

We emphasise significant deficiencies in the current legislative landscape that pose challenges to these vital nursing practices and call for urgent revisions of the legislative framework, particularly the revision of Section 56 of the *Nursing Act (33 of 2005)* and its related regulations, to formalise authorisation of specialist nurse prescribers in public and private practice. This will also entail an application to the South African Health Products Regulatory Authority (SAPHRA) for the scheduling of substances by authorised nurse prescribers in the defined professional nurse and specialist nurse categories by the Minister of Health.

**Conclusion:**

There is a necessity for prompt legislative revisions to address identified deficiencies.

**Contribution:**

The contribution of this article lies in its advocacy for changes to the regulatory framework to further enable nurses to deliver safe and comprehensive health care.

## Introduction

Nurse prescribing and dispensing have emerged as vital components of South Africa’s healthcare strategy, aimed at increasing accessibility, affordability, and efficiency. This discussion article explores the historical evolution of the legal enablements of nurse prescribing and dispensing in South Africa prior to and after 1994. The authors further identify critical legislative gaps that continue to compromise these essential nursing practices.

## Historical antecedents and current developments

The roots of the primary health care (PHC) approach can be traced to the 1940s in rural KwaZulu-Natal (Kautzky & Tollman 2008). The Pholela Health Centre model was a forerunner of community-orientated primary care (COPC) and a pioneering public health approach implemented by Dr Sydney and Emily Kark to address healthcare disparities and improve access to services. The model exemplified task shifting and sharing where health assistants and community educators facilitated the provision of health education and health promotion at a village and household level. Sadly, initiatives for a national health system reform were halted because of a lack of political will and financial support at the time. It appeared that the nursing profession also viewed the model as a threat (Kautzky & Tollman 2008).

Primary health care was resurrected during the apartheid era, specifically during the establishment of the homelands in the 1970s. The homelands were designated economically disadvantaged areas set aside for black South Africans based on their ethnic or tribal identities and enforced by the apartheid government to promote racial segregation. The 1976 Soweto uprising (student-led protests against the compulsory use of Afrikaans in black schools that led to violent clashes with the police) led to a shortage of medical officers at PHC clinics, creating a demand for task shifting to nurses and the development of a clinical skills training course. A new cadre of ‘PHC nurses’ was established who were authorised to assess and diagnose patients, as well as prescribe treatment and supply medication (Geyer 2001; Kautzky & Tollman 2008) Section 38A of the *Nursing Act* (*No. 50 of 1978*) provided for the authorisation of certain nurses to diagnose, prescribe, and supply medicines (Republic of South Africa 1978). A section 38A permit could be issued to a registered nurse employed by the national or provincial Departments of Health, local authorities, or any organisation performing a health service designated for this purpose by the Director-General of Health (Geyer 2001; South Africa 1978). A ‘nurse-clinician’ course was provided for (R. 48 of 1982, Regulations for the Diploma in Clinical Nursing Science, Health Assessment, Treatment and Care), and the keeping, supply, administering or prescribing of medicines by registered nurses were regulated (R. 2418 of 1984). These regulations limited nurses with section 38A permits to prescribing specific unscheduled medicines (now Schedule 0) and those in Schedules 1–4.

After the democratic transition in 1994, the PHC approach was adopted by the South African government to provide accessible and equitable healthcare to all (Barron & Roma-Reardon 2008). The 1997 White Paper on the Transformation of the Health System in South Africa committed to comprehensive PHC, delivered via a district health system (DHS). The National Drug Policy (1996), was added as an appendix to the 1997 White Paper and promoted rational prescribing and responsible dispensing practice to ensure that all health personnel involved in the diagnosis, prescribing, and dispensing of medicines receive adequate theoretical and practical training. In order to facilitate broader access to prescription medicines, a wider range of competent prescribers were recognised. In particular, the Minister of Health (1996) included the following statement: ‘At primary level prescribing will be competency, not occupation, based’.

Task shifting, a global strategy to optimise healthcare delivery, further bolstered the role of nurses in South Africa (WHO 2008). By redistributing tasks from medical practitioners to nurses, particularly in underserved and rural areas, the healthcare system could address the shortage of medical professionals and pharmacists and improve access to healthcare. In 2010, the need to rapidly scale up and decentralise the human immunodeficiency virus (HIV) programme in South Africa was a further impetus to expand the scope of practice of nurses through nurse-initiated and managed antiretroviral therapy (NIMART). A growing body of evidence indicated that nurse-led or non-physician-led models of care can be just as effective as physician-led models (Emdin, Chong & Millson 2013). With appropriate training, clinical guidelines, supervision and support, nurses were entrusted with prescribing and supplying antiretroviral therapy (ART), acknowledging the concept of task sharing (Okoroafor & Christmals 2023). Nurses, for instance, were empowered to commence first-line treatment in uncomplicated cases, while medical practitioners were responsible for overseeing and managing patients with more complex conditions. More recently, pharmacist-initiated management of antiretroviral therapy (PIMART) has continued this momentum, ensuring access to combination ART as well as HIV pre-exposure prophylaxis (PrEP). This led to a call to the South African Minister of Health to recognise overlapping scopes of practice and the need for prescribing to be competency-based and not occupation-based, in line with the National Drug Policy prescript, provided that clear guidelines and effective referral practices are in place (Moodley et al. 2021).

With the implementation of National Health Insurance (NHI) in South Africa, health care services are expected to continue to be decentralised to communities, with the provision of appropriate services at schools and workplaces. Nurses will continue to play an integral role in ensuring effective universal health coverage via school-based services, occupational health services, child health services, sexual reproductive health services, and communicable as well as non-communicable disease prevention and management. Many of these services involve prescribing and dispensing to some extent, in particular, immunisation, family planning, HIV and tuberculosis (TB) prevention and treatment, and the management of non-communicable diseases. Further, there is a growing need to decentralise the provision of mental health and palliative care services, which would require the provision of Schedule 5 and Schedule 6 medicines.

Despite these significant strides, challenges persist in the implementation of nurse prescribing and dispensing in South Africa, particularly related to the enabling legislative framework and the processes of ensuring safe nursing practice and patient care.

## Legislation governing nurse prescribing and dispensing

It is important to examine not only the primary legislation (the Acts) but also the secondary (Regulations) and, in certain instances, the tertiary (Board notices), in order to reveal any contradictions embedded within the legislation, thus offering a clearer understanding of its nuances and implications.

### The Nursing Act No. 33 of 2005 and Medicines and Related Substances Act No. 101 of 1965

The practice of nursing is currently governed by the *Nursing Act No. 33 of 2005* and its accompanying regulations. The *2005 Nursing Act* has been fully promulgated, replacing the *1978 Nursing Act* in its entirety. In the *2005 Act*, section 56 provides the regulatory framework for the prescribing and dispensing of medicines by nurses. Section 56(1) indicates that the South African Nursing Council (SANC) may register a professional nurse, midwife or enrolled nurse to assess, diagnose, prescribe treatment, keep and supply medication for prescribed illnesses and health-related conditions if such a person: (1) provides proof of completing prescribed training, (2) pays the required fees, and (3) complies with subsection 6. Subsection 56(5) specifies that acquiring, using, possessing or supplying and dispensing of medicine are subject to the *Medicines and Related Substances Act No. 101 of 1965* or ‘Medicines Act’.

Section 22(A)(4) and (5) of the *Medicines and Related Substances Act* (*101 of 1965*) states that medicines in Schedules 1–6 can be sold by:

‘[*A*] practitioner, nurse or a person registered under the Health Professions Act, 1974, other than a medical practitioner or dentist, who may [*aa*] prescribe only the Scheduled substances identified in the Schedule for that purpose; [*bb*] compound and dispense the Scheduled substances referred to in item [*aa*] only if he or she is the holder of a licence contemplated in section 22C[*1*][*a*].’

Further, section 22(A)(14) states that:

‘No nurse or a person registered under the Health Professions Act, 1974, other than a medical practitioner or dentist, may prescribe a medicine or scheduled substance unless he or she has been authorised to do so by his or her professional council concerned.’

The section 22A(14) requirement of formal authorisation from the professional council has created challenges for nurses to prescribe because there is no formal SANC authorisation process in place at present. In some instances, pharmacists refused to dispense the prescriptions of nurses in the absence of evidence of authorisation. Dispensing of medications is the primary role of pharmacy personnel and is regulated by section 22 of the *Medicines Act* and the regulations relating to the practice of pharmacy published in terms of the *Pharmacy Act* (*Act No. 53 of 1974*). A pharmacist may only dispense the prescription of an authorised prescriber.

Dispensing licences are issued by the Director-General in terms of section 22(C)(1)(a) of the *Medicines and Related Substances Act*. A dispensing licence can only be issued to a person who is an authorised prescriber, as recognised by their statutory council. Even though, ideally, the processes of prescribing and dispensing should be separated to ensure the safety of the patient, the prohibition is not absolute as medical practitioners are allowed by section 22C(1)(a) to be licenced to dispense after completing additional training (Moodley et al. 2021).

Section 22A(15) provides for an exceptional permit, stating:

‘Notwithstanding anything to the contrary contained in this section, the Director-General may, after consultation with the South African Pharmacy Council as referred to in section 2 of the Pharmacy Act, 1974 [*Act No. 53 of 1974*], issue a permit to any person or organisation performing a health service, authorising such person or organisation to acquire, possess, use or supply any specified Schedule 1, Schedule 2, Schedule 3, Schedule 4 or Schedule 5 substance, and such permit shall be subject to such conditions as the Director-General may determine.’

Section 22A(15) is broadly enabling and allows the Director-General to issue a permit to any person or organisation performing a health service. As a result, those with the permit may issue medicines listed in the permit and do not require dispensing licences. However, a nurse holding a section 22A(15) permit is not regarded as an authorised prescriber and cannot issue prescriptions for dispensing at pharmacies (SAPC 2022). Although nurses in private practice providing immunisation services, family planning, home-based care or haemodialysis services can apply for a section 22A(15) permit without the need for a dispensing licence, these applications hinge on certain requirements such as physical infrastructure to keep and store medications.

However, in subsection 56(6) of the *Nursing Act*, a concession is created, analogous to the previous section 38A. Any nurse working in the state sector, or a designated organisation may be issued a permit allowing physical assessment, diagnosis of a ‘physical defect, illness or deficiency’, keeping, supplying, administering, and prescribing medicines. Importantly, the law states that the issuing of these permits is ‘despite the provisions of the Medicines and Related Substances Act, 1965, the Pharmacy Act, 1974 (Act No. 53 of 1974), and the Health Professions Act, 1974 (Act No. 56 of 1974)’. In other words, this section is not subject to any of the restrictions imposed by the listed Acts. Such acts are intended to be performed by nurses only if the services of a medical practitioner or pharmacist are not available (Republic of South Africa 2005). Practically, to manage the number of patients presenting to health facilities, nurses with section 56(6) permits often work alongside medical and pharmacy personnel.

Regulations relating to the keeping, supply and administering, prescribing or dispensing of medications by registered nurses (Government Notice No. R. 1044) were published for public comment in 2011. However, these regulations were never finalised and included inappropriate and contradictory elements. Currently, the existing regulation (R. 2418 of 1984) issued in terms of the *1978 Nursing Act* to accompany section 38A is still in force, in accordance with the transitional provisions in the *2005 Nursing Act*. Section 61(1) specifies that regulations issued under the old Nursing Act stay in effect unless inconsistent with the new Act. Nurses can therefore only supply, administer or prescribe medicines containing substances listed in Schedule 0 to 4. The limitation of R. 2418 was already identified in 2001 by Geyer (2001) as creating a stumbling block for the psychiatric nurse and the nurse in palliative care. In contrast, section 22A of the *Medicines Act* makes provision for the nurse to be authorised to prescribe Schedule 1–6 medicines listed for that purpose in the Schedules, provided the nurse is deemed competent by the SANC. That provision can only be operationalised if the SANC prescribes the training for professional and specialist nurses, creates the registers for such categories, and liaises with the South African Health Products Regulatory Authority (SAHPRA) to ensure that medicines to be prescribed by nurses are listed for that purpose in the Schedules by the Minister of Health. Such nurses would need a section 22C(1)(a) dispensing licence, as signalled in section 56(5) of the *Nursing Act*.

The SANC has not yet indicated what the prescribed training should be in order to authorise nurses to prescribe, whether there will be different authorisations for different SANC registration categories, and if there will be specific medication lists and/or schedules linked to a particular authorisation. Therefore, the SANC does not currently authorise nurses to prescribe, as required by section 22A(14) of the *Medicines and Related Substances Act*. At the moment, nurses working for the state or designated organisations can be authorised under section 56(6) to prescribe medications, irrespective of whether they are registered nurses with a basic qualification (R. 425, R. 171 or R. 174) or have completed post-basic training as specified in R. 48 of 1982 or the new R. 635 of 2020 (Regulations relating to the minimum requirements for the education and training of a student leading to registration as a nurse specialist or midwife specialist). Although some guidelines such as the Department of Health Adult Primary Care (APC) guidelines and the Western Cape Practical Approach to Care Kit (PACK) guidelines have distinguished between medications that can be prescribed by a registered nurse and those that can be prescribed by a post-basic trained nurse or ‘Clinical Nurse Practitioner’, these have not been formalised legally.

There are limitations of section 56(6) that are similar to those of the old section 38A. These limitations were highlighted by Geyer (2001) and should be improved to clarify the interpretation:

1. Nurses in private practice cannot secure the necessary legal authorisation to prescribe medication unless they have some form of employment affiliation with a designated organisation.
2. It is assumed that section 56(6) also applies to midwifery practice. Midwives working in the public sector must rely on prescriptions issued by medical practitioners for individual patients, although this may not be the case in the midwife-led Midwife Obstetric Units (MOUs). However, midwives have a separate provision in the General Regulations (31) to the *Medicines Act* allowing access to analgesia listed in the Standard Treatment Guidelines and Essential Medicines List (STG/EDL), including Schedule 6.
3. Nurses employed by the Defence Force, Mines, and Correctional Services are excluded from the provisions in section 56(6) unless those organisations are designated by the Director-General of Health.
4. The current framework in section 56(6) only allows for ‘physical’ assessment and diagnosis, thereby excluding assessment of mental health disorders from the scope. While the immediate challenge revolves around obtaining authorisation for Schedule 5 and 6 medicines, it is essential to address this constraint for a more comprehensive and inclusive approach.
5. Authorisation is presently restricted to circumstances of doctor or pharmacist shortages, a stance conflicting with the government’s healthcare policy that encourages task sharing. Nonetheless, the current interpretation of this provision leans towards a more permissive stance.
6. Currently, there is a notable absence of SANC guidelines or regulations overseeing the authorisation process for nurses to diagnose and prescribe, despite the existence of a policy document from the National Department of Health.

Although in the past it was deemed necessary for nurses to practise in the grey area of overlapping scopes of practice only in the absence of medical practitioners and pharmacists, changes in the health system such as the recognition of task sharing, for example, in the case of NIMART since 2010, and the emergence of various specialist nursing programmes have necessitated a clear description of the role of nurses (and midwives) within prescribing and dispensing practice. It is therefore imperative that the SANC review and amend section 56 of the *Nursing Act* and publish accompanying regulations so that the process of authorisation to prescribe can be formalised and not rely primarily on the section 56(6) concession.

### South African National Department of Health guidance

The Director-General of the South African National Department of Health issued a circular (LU-CIR-022016/01) in 2016 to clarify that nurses in the service of provincial and municipal departments of health may only prescribe medication in accordance with STG/EDL for PHC, if they have been authorised to do in terms of section 56(6) by the head of the provincial department of health or the medical officer of the municipality or a person with delegated power.

Pharmacists may dispense a written prescription of a nurse authorised to prescribe if there is evidence that the nurse is duly authorised in accordance with section 56(6). The onus is on the pharmacist to validate that in terms of the *Medicines and Related Substances Act* (SAPC 2022). The Director-General called on provincial departments of health to maintain a current list of all nurses authorised in terms of section 56(6) that is made available to pharmaceutical services and accessible to pharmacists. A nurse in the public sector authorised in terms of section 56(6) does not require a dispensing licence or a section 22A(15) permit to prescribe medicine.

### The South African Nursing Council position

In a position statement related to prescribing and dispensing by nurses in July 2021, the SANC restated that section 56(6) is only applicable to nurses employed in the state or organisations designated by the Director-General. They further state that nurses employed at an organisation designated by the Director-General need to apply for a dispensing licence and comply with all relevant guidelines such as the Good Pharmacy Practice guidelines. Although the Department of Health circular states that nurses employed at designated organisations and have a section 56(6) authorisation do not need a dispensing licence, it appears to be the view of the SANC that they do, and employers expect these nurses to have a dispensing licence. Further, according to SANC, nurses who hold a ‘post basic’ qualification and are not employed by the state or designated organisations can be issued with a section 22A(15) permit, should they meet the requirements of the Department of Health, SANC, and Pharmacy Council (SANC 2021). The requirement of a post-basic and/or additional qualification is another discrepancy as it is not included in section 56(5) or the Department of Health guidelines. However, it appears that it is the view of the SANC that nurses should possess an additional qualification.

### South African health products regulatory authority guidance

The STG/EDL for PHC issued by the National Department of Health does not specify the prescriber level for each of the medicines and there is no official designation of any of the medicines as being nurse-initiated (SAHPRA 2022). The South African Health Products Regulatory Authority (SAHPRA 2022) has provided guidance related to the listing of medicines to be accessed by health professionals, including nurses, who are deemed competent to be authorised prescribers of medicines. An application can be made to SAHPRA to amend the Schedules in order to designate specific substances to be prescribed by selected health professionals. In the case of nursing:

1. There should be evidence of the particular category of nurse who will be considered by the statutory council concerned as being ‘authorised prescribers’ as outlined in section 22A(14)(b) of the *Medicines and Related Substances Act, 1965* (Act 101 of 1965). The category must be a holder of a specific registration after completing the required training provided by an accredited provider.
2. A description of the competencies held by such holders of registration, indicating the clinical conditions which would be appropriate to be diagnosed and managed by such persons.
3. A detailed description of the curriculum, the nature of the practical clinical training provided, as well as the approach to assessment of clinical competence.
4. A clear and justified listing of the substances to be included in Schedules 1–6 (as appropriate), linked to the list of conditions to be managed by such holders of registration.
5. Current registration with the SANC.

Applications will be considered by SAHPRA, and once resolved, a draft set of proposed changes to the Schedules will be published to allow for comment by interested persons and institutions. Thereafter, a final set of recommended changes will be submitted to the Minister of Health for publication in the Government Gazette, in terms of section 37A of the *Medicines and Related Substances Act, 1965* (Act 101 of 1965) (SAHPRA 2022).

While this opportunity to amend schedules has been embraced by emergency personnel, optometrists, podiatrists, dental therapists, and dental hygienists, there has been no movement for nurses. One major reason is the ‘concession’ provision in section 56(6).

### Ethical considerations

This article followed all ethical standards for research without direct contact with human or animal subjects.

## Implications and recommendations

The delayed response from the SANC in amending the *Nursing Act* and associated regulations could, in part, be attributed to a strategic approach, possibly aiming to afford the Department of Health a wider latitude in deploying nurses for PHC services. Keeping legislative frameworks up to date poses inherent challenges within the evolving healthcare landscape, given its intricate processes. Nevertheless, this delay may have adverse implications for both nursing professionals and the public. Urgency is paramount in prioritising the review of the legislative framework that will empower nurses to prescribe and dispense medication. As a starting point, the SANC should prioritise revising section 56 of the *Nursing Act* and take up the SAHPRA guidance to apply for the scheduling of substances for prescribing by authorised nurses in the various specialist nursing categories, based on specific competencies. The current state is continuing to create an unfair labour practice where not all nurses may be appropriately trained to prescribe and manage medicines under section 56(6). It further limits the scope of practice of nurses in private practice, unless they operate in an organisation designated by the Director-General.

## Conclusion

Nurse prescribing and dispensing have evolved as vital components of South Africa’s healthcare system, driven by a commitment to expand healthcare access and quality. Identifying and addressing current gaps can further empower the South African nursing workforce, ensuring that they can continue to play a pivotal role in shaping the future of healthcare in the nation.

## References

[CIT0001] Barron, P. & Roma-Reardon, J., 2008, ‘Editorial. Primary health care in South Africa: A review of 30 years since Alma Ata’, in P. Barron & J. Roma-Reardon (eds.), *South African health review 2008*, Health Systems Trust, viewed 24 September 2023, from https://www.hst.org.za/publications/Pages/HST-SOUTH-AFRICAN-HEALTH-REVIEW-2008.aspx.

[CIT0002] Emdin, C.A., Chong, N.J. & Millson, P.E., 2013, ‘Non-physician clinician provided HIV treatment results in equivalent outcomes as physician-provided care: A meta-analysis’, *Journal of the International AIDS Society* 16(1), 18445. 10.7448/IAS.16.1.18445 23827470 PMC3702014

[CIT0003] Geyer, N., 2001, ‘Enabling legislation in diagnosis and prescribing of medicine by nurses/health practitioners’, *Curationis*, November, pp. 17–24, viewed 24 September 2023, from https://curationis.org.za/index.php/curationis/article/download/873/810#:~:text=The%20limitations%20of%20section%2038A,a%20designated%20or%20%2Dganization%20exists. 10.4102/curationis.v24i4.873 11993258

[CIT0004] Kautzky, K. & Tollman, S.M., 2008, ‘A perspective on primary health care in South Africa’, in P. Barron & J. Roma-Reardon (eds.), *South African health review 2008*, Health Systems Trust, viewed 24 September 2023, from https://www.hst.org.za/publications/South%20African%20Health%20Reviews/2%20A%20Perspective%20on%20Primary%20Health%20Care%20in%20South%20Africa%20SAHR%202008.pdf.

[CIT0005] Minister of Health, 1982, *Government Notice No. R. 48 (as amended)*, South African Nursing Council Regulations for the Diploma in Clinical Nursing Science, Health Assessment, Treatment and Care, viewed 30 July 2023, from https://www.sanc.co.za/r-48/.

[CIT0006] Minister of Health, 1996, National Drug Policy for South Africa, p. 18, viewed 24 March 2024, from https://www.sapc.za.org/Media/Default/Documents/Reference%20-%20National%20Drug%20Policy%20for%20South%20Africa.pdf.

[CIT0007] Minister of Health, 2011, *Government Gazette No. R1044, 11 Dec 2011, Regulations related to the keeping, supply and administering, prescribing or dispensing of medications by registered nurses*, viewed 30 July 2023, from https://www.gov.za/sites/default/files/gcis_document/201409/34851rg9646gon1044.pdf.

[CIT0008] Minister of Health, 2020, *Government Gazette No. R635 of 5 June 2020. Regulations relating to the minimum requirements for the education and training of a student leading to registration as a nurse specialist or midwife specialist*, viewed 25 September 2023, from https://www.gov.za/sites/default/files/gcis_document/202306/48696gen3489.pdf.

[CIT0009] Moodley, S., Gray, A., Schellack, N., Schellack, N., Venter, F., Venter, F. et al., 2021, ‘Pharmacist-initiated antiretroviral therapy (PIMART)’, *South African Medical Journal* 111(12), 1162–1163. 10.7196/SAMJ.2021.v111i12.16262 34949299

[CIT0010] Okoroafor, S.C. & Christmals, C.D., 2023, ‘Task shifting and task sharing implementation in Africa: A scoping review on rationale and scope’, *Healthcare (Basel, Switzerland)* 11(8), 1200. 10.3390/healthcare11081200 37108033 PMC10138489

[CIT0011] Republic of South Africa, 1965, *Government Gazette, Medicines and Related Substances Amendment of Section 1 of Act No 101 of 1965*, viewed 30 July 2023, from https://www.gov.za/sites/default/files/gcis_document/201409/32148434.pdf.

[CIT0012] Republic of South Africa, 1974, *Government Gazette*, as amended, *Pharmacy Act No. 53 of 1974*, viewed 30 July 2023, from https://www.sahpra.org.za/wp-content/uploads/2022/07/Pharmacy-Act-53-1974.pdf.

[CIT0013] Republic of South Africa, 1978, *Nursing Act No 50 of 1978*, viewed 24 March 2024, from https://www.sahpra.org.za/document/nursing-act-1978-act-no-50-of-1978/.

[CIT0014] South African Nursing Council, 2005, *Nursing Act No. 33 of 2005*, viewed 30 July 2023, from https://www.sanc.co.za/wp-content/uploads/2020/06/Nursing-Act-2005.pdf.

[CIT0015] South African Health Products Regulatory Authority, 2022, *SAHPGL-CEM-NS-04_v3_Scheduling of substances for prescribing by authorised prescribers other than medical practitioners or dentists*, viewed 24 September 2023, from https://www.sahpra.org.za/wp-content/uploads/2022/05/SAHPGL-CEM-NS-04_v3-Scheduling-of-Substances-for-Prescribing-by-Authorised-Prescribers-other-than-Medical-Practitioners-or-Dentists.pdf.

[CIT0016] South African National Department of Health, 2016, *Circular. Prescribing by nurses*, Reference: LU-CIR-022016/01.

[CIT0017] South African Nursing Council, 2021, *Position statement relating to assessing, diagnosing of patients and the related keeping, supply and prescribing of medicines by nurses*, viewed 30 July 2023, from https://www.sanc.co.za/wp-content/uploads/2021/08/Position-Statement-Assessing-diagnosing-of-patients-and-the-related-keeping-supplying-and-prescribing-of-medicines-by-nurses.pdf.

[CIT0018] South African Pharmacy Council, 2022, *SAPC e-note: Practice advisory: Prescribing nurses*, viewed 30 July 2023, from https://www.pharmcouncil.co.za/Media/Default/Documents/Stakeholder%20Communication/Binder1.pdf.

[CIT0019] World Health Organization, 2008, *Task shifting – Rational redistribution of tasks among health workforce teams: Global recommendations and guidelines*, viewed 30 July 2023, from https://apps.who.int/iris/bitstream/handle/10665/43821/9789?sequence=1#:~:text=Task%20shifting%20is%20the%20name,shorter%20training%20and%20fewer%20qualifications.

